# Recombination mapping of the Brazilian stingless bee *Frieseomelitta varia* confirms high recombination rates in social hymenoptera

**DOI:** 10.1186/s12864-021-07987-3

**Published:** 2021-09-18

**Authors:** Prashant Waiker, Fabiano Carlos Pinto de Abreu, Danielle Luna-Lucena, Flávia Cristina Paula Freitas, Zilá Luz Paulino Simões, Olav Rueppell

**Affiliations:** 1 Biology Department, University of North Carolina at Greensboro, 321 McIver St, Greensboro, NC 27412 USA; 2 Departamento de Biologia, Faculdade de Filosofia, Ciências e Letras de Ribeirão Preto, Universidade de São Paulo, SP Ribeirão Preto, Brazil; 3 Departamento de Genética, Faculdade de Medicina de Ribeirão Preto, Universidade de São Paulo, Ribeirão Preto, SP Brazil; 4 Departamento de Biologia Celular e do Desenvolvimento, Instituto de Ciências Biomédicas, Universidade Federal de Alfenas, Alfenas, MG Brazil; 5 Department of Biological Sciences, University of Alberta, AB T6G 2E9 Edmonton, Canada

**Keywords:** Social evolution, Meiotic recombination, Stingless bee, Hymenoptera, Sociality, Genome evolution

## Abstract

**Background:**

Meiotic recombination is a fundamental genetic process that shuffles allele combinations and promotes accurate segregation of chromosomes. Analyses of the ubiquitous variation of recombination rates within and across species suggest that recombination is evolving adaptively. All studied insects with advanced eusociality have shown exceptionally high recombination rates, which may represent a prominent case of adaptive evolution of recombination. However, our understanding of the relationship between social evolution and recombination rates is incomplete, partly due to lacking empirical data. Here, we present a linkage map of the monandrous, advanced eusocial Brazilian stingless bee, *Frieseomelitta varia*, providing the first recombination analysis in the diverse Meliponini (Hymenoptera, Apidae).

**Results:**

Our linkage map includes 1417 markers in 19 linkage groups. This map spans approximately 2580 centimorgans, and comparisons to the physical genome assembly indicate that it covers more than 75 % of the 275 Megabasepairs (Mbp) *F. varia* genome. Thus, our study results in a genome-wide recombination rate estimate of 9.3–12.5 centimorgan per Mbp. This value is higher than estimates from nonsocial insects and comparable to other highly social species, although it does not support our prediction that monandry and strong queen-worker caste divergence of *F. varia* lead to even higher recombination rates than other advanced eusocial species.

**Conclusions:**

Our study expands the association between elevated recombination and sociality in the order Hymenoptera and strengthens the support for the hypothesis that advanced social evolution in hymenopteran insects invariably selects for high genomic recombination rates.

**Supplementary Information:**

The online version contains supplementary material available at 10.1186/s12864-021-07987-3.

## Background

Meiotic recombination is a universal process in sexual organisms that facilitates accurate segregation of chromosomes, which is achieved by the physical connection between homologous chromosomes. This connection depends on the formation of at least one reciprocal exchange between homologous chromosomes, a crossover [1]. In most eukaryotes, these crossover events occur once or twice per chromosome pair during meiosis [2]. The narrow range is presumably a consequence of a rather invariant selection for a minimal number of crossovers that are required to avoid aneuploidy while minimizing the risk of genomic instability or other deleterious effects of recombination [3]. However, recombination also allows for a reciprocal exchange of genetic material, facilitating adaptive evolution [4]. Explanations of these evolutionary benefits include the reduction of Hill-Robertson interference [5], the “Red Queen” hypothesis [6], and avoidance of Muller’s rachet [7]. The process of meiotic recombination increases the efficiency of natural selection by shuffling allele combinations in offspring and can create a greater genotypic variation that selection can act upon [8]. Based on these evolutionary arguments, the recombination rate is predicted to vary more widely than what is structurally required. Accordingly, recombination rate varies significantly across species, populations, and individuals [9–11], in addition to local variation within genomes [12]. Some of this variation can be linked to directional selection and environmental fluctuation, while some may be non-adaptive, and yet other variation may be reported due to measurement errors [12–16].

The high recombination rates of social Hymenoptera present a prominent case of recombination rates that are above the minimally required crossover numbers to guarantee proper chromosome segregation [17–22]. Reports of high recombination rates in all studied social hymenopteran species – four honey bees, two ants, one wasp, and one bumblebee – support this notion when compared to the lower recombination rates of solitary hymenopterans [19, 23]. Social evolution in the order Hymenoptera has led repeatedly to highly complex societies with reproductive division of labor, cooperative brood care, and overlapping generations [24]. Social insects vary in social complexity [25], and the level of social complexity may be related to recombination rate [20, 26].

Current hypotheses to explain the high recombination rates of social insects can be principally divided into several arguments. The first set is based on a short-term evolutionary advantage of recombination by increasing genotypic diversity to enhance disease resistance, division of labor, or potentially other factors [18, 19, 23]. Genetic diversity arguments have also been proposed to explain multiple mating by females (polyandry) in social Hymenoptera [27] and supported by numerous empirical studies [28, 29]. However, modeling indicates that polyandry leads to a much stronger increase in offspring genetic diversity than any recombination effect and therefore it has been argued that increased recombination is unlikely to have evolved by selection on colony genetic diversity [30]. The second argument is based on the idea that increased recombination rates have been selected for to facilitate the rapid, independent evolution of caste-specific genes in social insects and allow the evolution of caste differences [31]: High recombination may facilitate the divergence of queen and worker phenotypes, especially when worker- and queen- selected genes are physically close [32]. Correlations between recombination rate and the location of genes that are important for caste-specific functions support this argument [31, 33]. However, these correlations have not been consistently found even in the same species *Apis mellifera* [34], making this argument contentious [23]. A third argument, the potential for high genomic recombination to reduce the potential for kin conflict [35, 36] and selfish genetic elements [37] in social insects, is also plausible. The lack of a clear correlation between chromosome number and sociality [38] argues against this “reduction of genetic conflict” hypothesis, but selfish genetic elements enabled by reduced recombination certainly exist in the form of “social chromosomes” in genetically heterogeneous social insect societies [39]. Thus, more empirical data are needed to evaluate the validity of these theoretical arguments.

In addition to their high genomic recombination rates, the socially complex ant, wasp, and honey bee species share important sociobiological features [40]. All of these species are polyandrous even though monandry was the ancestral state in each clade [41]. While polyandry may indicate selection for genetic diversity within colonies, recombination and polyandry may both increase genotypic diversity. Thus, we are predicting that monandrous species with advanced eusociality exhibit higher recombination rates than comparable polyandrous species if recombination is selected to increase intra-colonial genetic diversity. However, this prediction has not yet been tested. Furthermore, colonies of all investigated species contain only one reproductive queen, which is physically diverged from the worker castes. Nevertheless, workers have retained a functional ovary in all these species, indicating that queen-worker divergence is not as complete as in species with completely sterile workers. Based on a stronger divergent selection between worker- and queen-specific genes in species with complete worker sterility, such species are predicted to exhibit particularly high recombination rates based on the second of the above hypotheses.

An important taxon of social insects that has not yet been investigated regarding genomic recombination rates is the stingless bees (Meliponini), which make up the most specious tribe in the Apinae [42]. Stingless bees exhibit advanced eusociality and include several species that are essential pollinators in tropical ecosystems [43]. The pantropical distribution of stingless bees suggests that their origin dates back to the ancient Gondwana supercontinent more than 100 million years ago [44]. Despite their ecological relevance and biodiversity of about 600 described species in 60 genera, stingless bees remain understudied in all aspects, including their social behavior and genomic features [45, 46], such as recombination. This deficit contrasts particularly with honey bees, which only represent one genus of ten species that evolved during the past 25 million years [47]. Multiple studies within and across species of honey bees document their exceptional recombination rates, ranging from 17.4 to 37.0 centimorgan (cM) per megabase (Mb) [17, 21, 22, 33, 48, 49]. Stingless bees have presumably diverged from honey bees over 80 million years ago, and it is unclear how social their common ancestor was [42]. Stingless bees rival honey bees in social complexity, are predominantly monandrous, and can have completely sterile workers [50–53]. Thus, based on both, genotypic diversity and caste divergence model, they are predicted to exhibit even higher recombination rates than honey bees. Stingless bees are also the most important missing taxon to assess the link between eusociality and high recombination rates in the Hymenoptera.

*Frieseomelitta varia* (Lepeletier, 1836) is a Neotropical, medium-sized species of stingless bee that occurs in several parts of Brazil [54]. Living in large colonies with one monandrous queen, *F. varia* workers are completely sterile with heavily modified and non-functional ovaries [50]. The genome of *F. varia* has been sequenced and assembled [55]. Hence, we chose *F. varia* to construct a high-quality recombination map using SNP markers, benefitting from recent advances in sequencing technology and the large number of haploid sons produced by a single female in this species.

## Results

The sequencing of genomic DNA of 180 *F. varia* males from a single mother resulted in highly variable numbers of high-quality reads ranging from 60,427 to 48,610,455 (mean: 13,428,476 ± 10,517,236 SD). A draft genome sequence for this mapping population was created from two individuals with highest read counts. These data from two individuals proved sufficient for a 301 Mbp assembly with an average GC content of 37 % (Table 1). This assembly was used as a direct reference for SNP calling to only discover the SNPs that were segregating in our mapping population. However, for downstream analyses the published, more contiguous genome Fvar_1.2 [55] was used. On average, 81 % (± 0.03 % SD) of the reads from each sample aligned to this reference (Table S1), and 9514 SNP markers were extracted after preliminary quality filtering.

**Table 1:** Genome assembly of *Frieseomelitta varia* used in this study for SNP calling (Sequences deposited in NCBI BioProject accession number PRJNA668370)

Statistics	Value
# scaffolds	102,310
Maximum Scaffold length	250.165 KB
Number of scaffolds > 50 KB	556
N/L50	4687/16.294 KB
N/L90	25,945/1.141 KB
GC (%)	36.93
Total genome length	301.35 MB

**Fig. 1:**
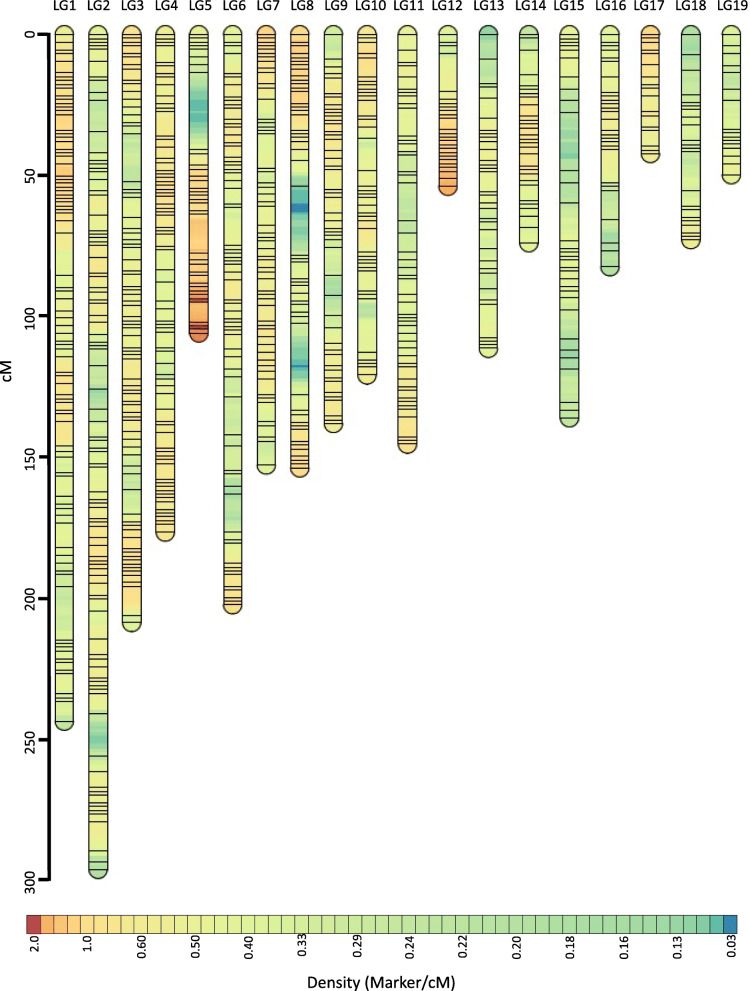
Linkage map of *F. varia*. The final genetic length of the map was 2558 cM, which consisted of 19 linkage groups ranging from 42 cM to 295 cM. The linkage groups are sorted according to descending marker numbers from 158 markers in LG1 to 18 markers in LG19. Further efforts to end-join any of these linkage groups failed. Each horizontal black line indicates an SNP marker, and their vertical position indicates recombination distances among markers. The color depicts approximate marker density within linkage groups. For complete plot with marker labels and positions please refer Supplementary Fig. 1

After filtering the original set of 9514 markers as described in methods, 1023 unique SNP markers were included in constructing the initial linkage map. The resulting map contained 20 linkage groups ranging from 49.5 cM to 242.9 cM, totaling 2573.2 cM. Post-hoc addition of previously excluded markers joined ends of two linkage groups but did not close any remaining linkage gaps (> 20 cM). The final map of *F. varia* comprised 1417 high-quality SNP markers assembled into 19 linkage groups (Fig. 1). Thus, the map is containing 4 extra linkage groups compared to 15 previously reported chromosomes [56]. The final map length was 2557.9 cM with linkage groups ranging from 42.0 cM to 295.4 cM. The average marker density was 0.55 markers per cM. The highest density was observed for group 12 (one marker every 1.03 cM), whereas group 15 had the lowest density (one marker every 3.31 cM) (Table 2). Based on a physical genome size of 275 Mb [55], the genetic map length resulted in a minimum estimate of 9.3 cM/Mb for the genome-wide recombination rate of *F. varia*.

**Table 2:** Summary of linkage groups of the *Frieseomelitta varia* map

Linkage group	Length (in cM)	Marker number	Marker density (Avg. cM distance between two consecutive markers)	# matched scaffolds	Combined scaffold length (in Mb)	Average LG rec. rate (LGRR) (in cM/Mb)
1	242.85	158	1.54	50	19.49	12.46
2	295.44	141	2.1	64	20.10	14.70
3	208.04	120	1.74	49	17.97	11.58
4	175.95	113	1.56	47	18.37	9.58
5	105.55	101	1.05	33	6.87	15.37
6	201.73	100	2.02	44	14.74	13.69
7	152.31	91	1.67	41	14.31	10.65
8	153.26	86	1.78	31	13.96	10.98
9	137.55	74	1.86	27	12.80	10.75
10	120.38	74	1.63	31	8.19	14.69
11	144.90	65	2.23	28	13.01	11.13
12	53.62	52	1.03	17	5.00	10.72
13	110.98	45	2.47	21	10.41	10.66
14	73.63	43	1.71	14	4.31	17.09
15	135.58	41	3.31	17	7.76	17.47
16	82.09	36	2.28	15	6.04	13.59
17	42.02	34	1.24	11	2.53	16.59
18	72.55	25	2.9	11	4.34	16.73
19	49.49	18	2.75	12	4.62	10.71
All	**2557.92**^b^	**1417**^b^	**1.81**^a^	**563**^b^	**204.82**^b^	**12.49**^a^

^a^Weighted average

^b^Sum

**Fig. 2:**
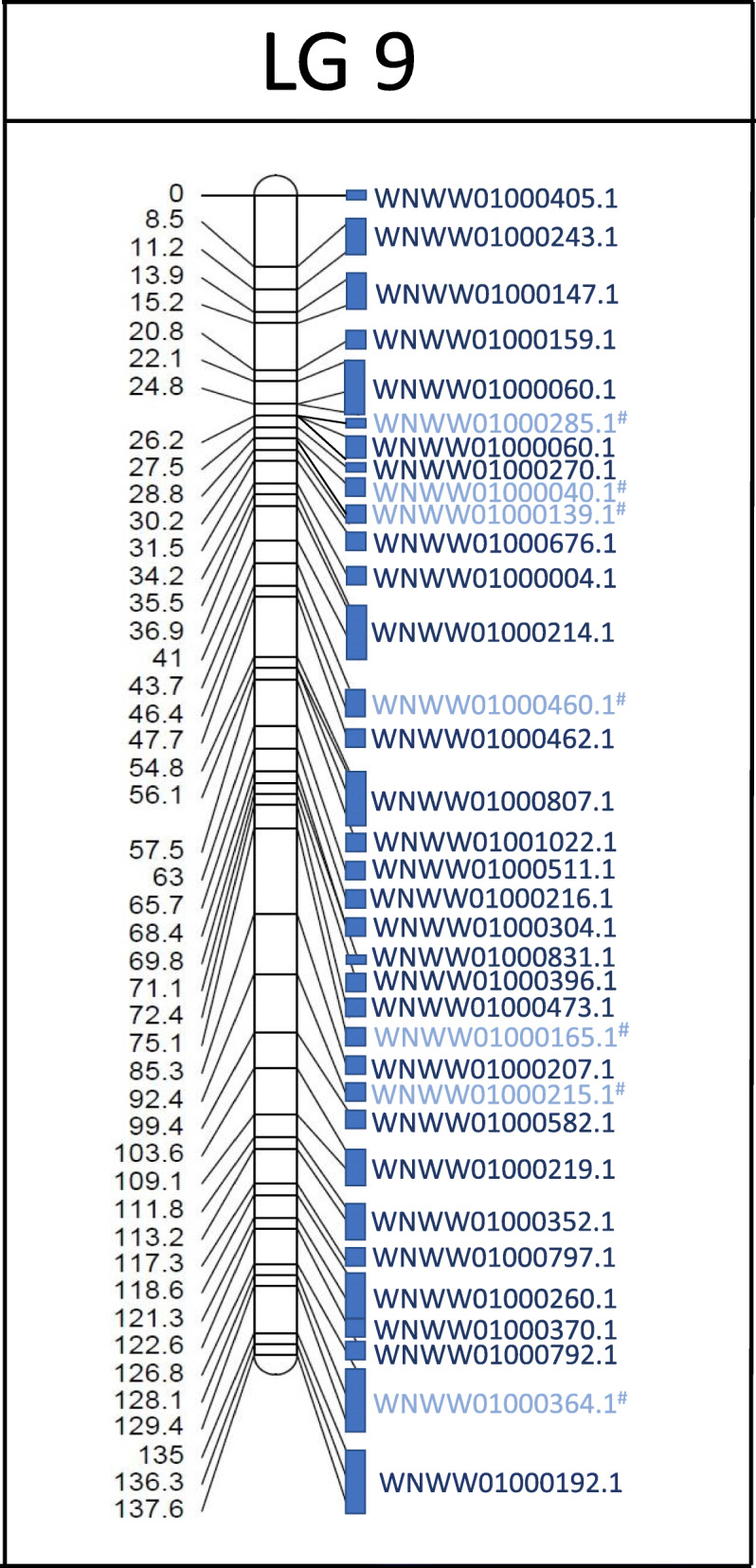
Example of genomic scaffold alignment to the linkage map of *F. varia*. Most of the genome scaffolds (Fvar_1.2) matched to sequences associated with SNPs of a single linkage group and had no match with other groups (indicated by dark blue text). However, some scaffolds matched to more than one linkage group (highlighted in light blue text color and ^#^). Blue boxes represent the approximate size of the matching scaffolds

The location of 99.1 % of the mapped SNPs was identified in Fvar_1.2 based on best nBLAST results (Table S2). The SNP sequences mapped to 563 unique scaffolds (25.9 % of all scaffolds) with a combined length of 204.8 Mb, representing 74.5 % of the total genome (Table 2). Scaffolds that were covered by our linkage map were much larger (median length = 260,348 bp) than scaffolds that were not represented (median = 4141 bp). The alignment of scaffolds to our linkage map uniquely mapped 451 scaffolds to our 21 linkage groups while 112 scaffolds mapped to multiple linkage groups (Fig. 2 and Figure S1). Based on the overall genome coverage of 74.5 %, the maximum estimate for the genome-wide recombination rate of *F. varia* equals 12.5 cM/Mb (2558 cM / 275 Mb * 0.745). On the basis of the length of the matching scaffolds, the average recombination rate for each linkage group was calculated, ranging from 9.6 to 17.5 cM/Mb (Table 2). A negative relationship between the average recombination rate of linkage groups and their physical length was observed, but this was not significant (Spearman’s correlation, R= -0.38, *n* = 19, *p*-value = 0.11; Figure S2).

## Discussion

Genome-wide recombination rates in social Hymenoptera are among the highest known in the Metazoa, but the molecular and evolutionary causes for this phenomenon remain unclear. Here, we present an additional case of high recombination in the highly social stingless bee *Frieseomelitta varia*, representing the diverse and important tribe Meliponini. This result represents a significant expansion of the correlation between advanced sociality and elevated recombination rates because stingless bees have diverged from honey bees over 80 million years ago [42, 47]. In addition to its taxonomic relevance, *F. varia* is significant because it exhibits monandry and completely sterile workers [50, 51], in contrast to all other social insects studied for recombination rates so far. The independently assembled genome of *F. varia* [55] allowed us to assess genome coverage of our linkage map to further refine the recombination estimate. However, both available assemblies were highly fragmented (Our assembly: Scaffolds = 102,310 and N50 = 4687; Fvar_v1.2 assembly: Scaffolds = 2173 and N50 = 470,005) and therefore deemed insufficient for meaningful analyses of genomic correlates of local recombination rates, as in species with higher-quality genomic resources [23, 31, 33].

The 19 linkage groups of our linkage map did not match the haploid chromosome number of 15 [56]. This difference could result from a lack of high-quality markers in certain genomic regions in our study, leading to incomplete genome coverage. Alternatively, the cytological determination of the number of chromosomes could be incomplete because small chromosomes can be easily missed in species with numerous chromosomes. A recent discovery of haploid number of 17 in another *Frieseomelitta* species supports this notion [57]. However, six of our linkage groups are smaller than the theoretical lower size limit of 100 cM (corresponding to one obligate crossover), which suggests that our linkage map is truly unsaturated. Given that over six-thousand markers were included in our analysis, this conclusion is surprising, but a systematic lack of sequencing results in AT-rich regions [58] could be responsible. The unsaturated map underestimates the actual genetic length by at least 120 cM (considering 30 cM depicts non-linkage between two groups, and we have potentially 4 excess groups). A corresponding adjustment increases the total genetic length of our linkage map to 2678 cM, resulting in a recombination estimate of 9.73 cM/Mbp.

The interpretation that our map is unsaturated is further supported by the comparison with the published genome “F_var1.2” [55]. However, each genomic scaffold that is not covered by our linkage map could be located in-between markers or represent true coverage gaps. Our markers cover about 25 % of the scaffolds and 74 % of the genome sequence. The missing scaffolds were generally shorter than the ones covered by our markers, indicating that these smaller scaffolds could indeed be located in the intervals between markers mapping to adjacent larger scaffolds. However, about 15 % of missing scaffolds were larger than 100 kbp, and these are less likely to be located within the existing linkage groups. As an upper estimate, we thus used the missing 25 % coverage of the sequenced genome to correct our total linkage map length and consequently genome-wide recombination estimate to 12.5 cM/Mbp.

Most of the mapped scaffolds correspond to unique linkage groups. However, 112 scaffolds were not unambiguously mapped to one linkage group due to conflicting nBLAST matches for their markers. This discrepancy between our linkage map and Fvar_v1.2 genome assembly may be due to inaccurate linkage mapping, nBLAST ambiguity due to sequence similarities of different genome locations, or problems in the genome assembly. Further discrepancies between the linkage map and the physical marker location were identified with respect to local marker order in a few scaffolds. Local marker ordering for linkage map construction can be error-prone when missing genotypes are incorporated. Our very stringent data exclusion standards have minimized the problem due to missing data but diminished our sample size and thus statistical power to infer the correct local marker order. This interpretation is supported by our finding that 222 markers belonging to different genome scaffolds were not separated by any recombination event in our data. With increasing sample size, the physical distance would eventually translate into a certain, although potentially small, recombination fraction. For our genome-wide recombination estimate, this sampling problem represents a conservative error, and the estimate might have to be further corrected upwards.

The number of chromosomes can itself impact genome-wide recombination rate, and the association between chromosome number and sociality has been tested with mixed results [35, 38]. With at least 15 chromosomes, *F. varia* has a high number of chromosomes, and this contributes to a high genome-wide recombination rate if we assume at least one recombination event per chromosome. However, most linkage groups exceeded the corresponding value of 100 cM, and the smaller groups likely have to be combined. The recombination rate estimates of our linkage groups were decreasing with the physical length of the corresponding genome sequence. However, this negative trend was not significant. Thus, the theoretically predicted relation may not exist in *F. varia*, particularly considering that some of the smaller linkage groups may, in fact represent fractions of large chromosomes. The absence of a negative relation between chromosome size and recombination rate has also been observed in *Apis mellifera* [17, 33] and maybe another indication for selection of recombination in excess of the structurally required minimum.

**Fig. 3:**
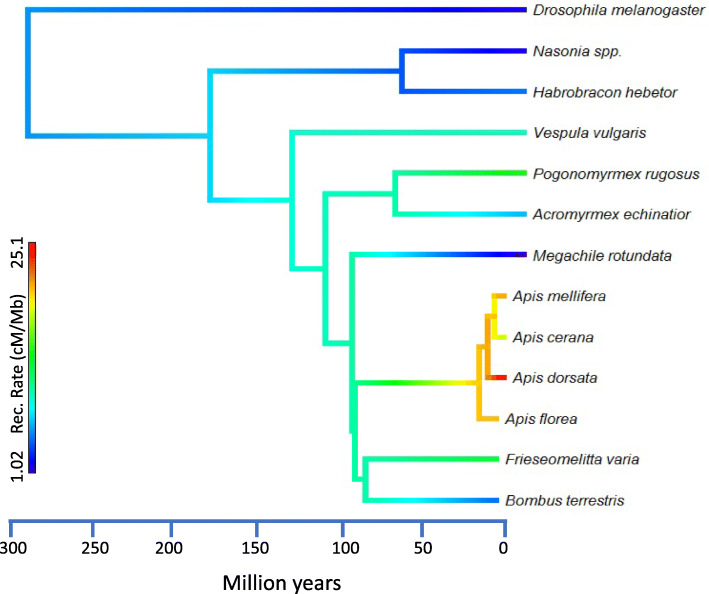
Recombination rate evolution in Hymenoptera. Recombination rates in the solitary outgroup (*Drosophila melanogaster* (1.6 cM/Mb), as well as solitary Hymenoptera *Nasonia* spp (1.5 cM/Mb), *Habrobracon hebetor* (4.8 cM/Mb*), and Megachile rotundata* (1.0 cM/Mb*)* are generally low, while advanced eusocial species always exhibit higher estimates, including the newly studied stingless bee *Frieseomellita varia* (12.5 cM/Mb). However, estimates in honey bees remain particularly high (*Apis florea*: 20.8 cM/Mb, *A. mellifera*: 21.6 cM/Mb, *A. cerana*: 17.4 cM/Mb, and *Apis dorsata*: 25.1 cM/Mb*)*, even when compared to other eusocial Hymenoptera, including ants (*Acromyrmex echinatior*: 6.4 cM/Mb and *Pogonomyrmex rugosus*: 14.0 cM/Mb), Bumble bees (*Bombus terrestris*: 8.9 cM/Mb), and wasps (*Vespula vulgaris*: 9.7 cM/Mb). The horizontal axis depicts approximate time, illustrating divergence between species. Recombination rate data sources: [18–23, 34, 40, 61, 62]. The tips of the tree are colored according to recombination rate estimates. ML ancestral states at each node are estimated by fastAnc() function of Phytools and colored on the same gradient [63]

Although the evolutionary conservation of extremely high recombination rates in the honey bee genus was established previously [22], based on our results, we cannot exclude the possibility that significantly elevated recombination rates may have originated before the evolution of honey bees and been evolutionarily conserved since the divergence of stingless bees, bumble bees and honey bees (Fig. 3) about 80 million years ago [59]. The recombination rates of orchid bees (Euglossini), the solitary sister taxon of honey bees [60], is unknown. A high recombination rates of orchid bees would strengthen an ancestral origin of high recombination. In contrast, low recombination rates in the Euglossini, as predicted based on the solitary lifestyle, could indicate an evolutionary reduction of recombination rates in this taxon or multiple independent origins of elevated recombination rates in honey bees, bumble bees and stingless bees in accordance with eusociality [60].

Compared to honey bees, our recombination rate estimates for *F. varia* are lower, regardless of species and methodology [17, 21, 22, 33, 34]. This finding contrasts with the prediction of a higher rate in *F. varia*, based on either its monandry or its social complexity. Our results do not support the view that high recombination compensates for monandry, which reduces genetic diversity compared to polyandry. Thus, our results do not support genetic diversity arguments for the evolution of high recombination in social insects. Similarly, the stronger caste divergence of *F. varia* compared to honey bees [50] and a specialized soldier caste [64] do not coincide with a higher recombination rate, as predicted by our second hypothesis. In contrast, the high recombination rates of *Apis* and the less elevated rates of *F. varia* and other social Hymenoptera may provide some support for the “reduced genetic conflict” hypothesis: Selection for homogenizing genetic relatedness [36] and against selfish genetic elements [37] is stronger in polyandrous than monandrous species [65], and honey bees exhibit not only exceptional recombination rates, but also an exceptional degree of polyandry [66]. The absence of ovaries in workers of *F. varia* is an additional factor that might reduce intra-colonial conflict compared to honey bees [50].

## Conclusions

Our genome-wide recombination rate estimate of 9.3–12.5 cM/Mb for the stingless bee *Frieseomelitta varia* adds an important case study to the growing list of social insect species that exhibit more frequent meiotic recombination than their non-social counterparts. *F. varia* represents an independent taxon and indicates that elevated recombination rates in social insects are consistent, even though this species differs from previously studied social insects in regards to important life-history variables. Our study thus corroborates the association between high recombination rates and sociality in the Hymenoptera, although more comprehensive tests across many social taxa need to be performed, and our understanding of the proximate and ultimate causation of this association remains incomplete.

This study presented the the genomic recombination rate of a representative species of the important, highly social taxon Meliponini. With an estimate between 9.3 and 12.5 cM/Mb, we corroborate the association between high recombination rates and sociality in the Hymenoptera. This result strengthens the argument that advanced social evolution in social hymenopterans selects for high genomic recombination rates. Contrasting our new estimate to the consistently higher values of honey bees highlights the need for more empirical and theoretical work on the evolution of recombination in social insects.

## Methods

### Sampling, DNA extraction, and Sequencing

*Frieseomellita varia* (Lepeletier) haploid males from a single mother were obtained from one colony from the southeast region of Brazil (Departamento de Genética Faculdade de Medicina de Ribeirão Preto, geographical coordinates: 21°10’12.2"S 47°51’34.2"W) between November 2018 and January 2019. The specimens were collected within the colony, kept in a glass vial on ice for about 5 min. Sex determination was based on presence of sexually dimorphic characters and gonads with the aid of a stereo microscope. To ensure that all offspring came from a single mother, the queen of the colony was color marked on the thorax (Posca Posta Pens, Japan) at the beginning of the experiment. During the collection period, no replacement of the queen was observed. Total genomic DNA was extracted from the whole body of 180 collected male offspring using the Wizard Genomic DNA Purification protocol (Promega, Dübendorf, Switzerland). The purity and concentration of extracted DNA were measured using a NanoDrop™ 1000 Spectrophotometer (Thermo Fisher Scientific, Wilmington, DE). Furthermore, DNA integrity was assessed by visually inspecting samples after gel electrophoresis (1.5 % agarose, 1X SB Buffer).

From each sample, 200 ng of DNA was sent to the SNPsaurus™ sequencing facility (Eugene, Oregon) for SNP genotyping by whole-genome resequencing. In short, genomic DNA was converted to Illumina sequencing libraries with a partial Nextera DNA Flex™ reaction (SNPsaurus, Eugene, OR) and sequenced on a NovaSeq 6000 S4™ (Illumina Inc, San Diego, CA) lane with paired-end 150 bp reads. Sequence reads were quality filtered, and adaptors trimmed with bbduk (BBtools, Bushnell B. – sourceforge.net/projects/bbmap) using trim parameters: ktrim = r k = 17 hdist = 1 mink = 8 minlen = 100 qtrim = rtrimq = 10pigz = t unpigz = t ordered = t. The trimmed reads from two samples, FV116 and FV89, were combined to create a draft genome assembly with abyss-pe [67] with default parameters. Reads from each sample were aligned to this draft assembly using bbmap (BBtools) using alignment parameters: minid = 0.95 ambig = toss k = 13 idtag maxindel = 30 | samtools view -bSu - | samtools sort -@64 –o sort_file. The aligned reads were converted to a VCF format genotype using callvariants (BBtools) using callvariant parameters: ploidy = 2 multisample = t nopassdot = f minavgmapq = 15 minreadmapq = 15 strandedcov = t. Variants that were identified and their surrounding 150 bp sequence were used as our SNP markers for linkage mapping as described below.

### SNP Filtering and Linkage mapping

The VCF file containing high-quality SNPs and Indels was filtered before linkage map construction based on the following criteria using VCFTools [68]: all SNPs with > 50 % missing data were removed (--max-missing 0.5); SNPs with a quality score < 30 were filtered out (--minQ 30); SNPs with a minor allele count of 3 or less were removed (--mac 3); and SNPs with a read depth of < 6 were excluded (--minDP 6). All VCFTools filtering command lines can be found in the supplementary methods (File S1). Subsequently, the 76 individuals with the least missing data (< 2 %) were chosen to generate a linkage map. Initial grouping at LOD 8 resulted in 32 linkage groups. 106 markers that were unlinked or linked to only one other marker were discarded. A total of 9404 SNPs was left after this filtering step. The markers with more than one missing data point in this refined dataset were excluded in a final filtering step, leaving 3556 SNP markers for final linkage map construction.

The 3556 SNP markers were duplicated, and the doubled set was assigned the opposite phase for mapping ‘Phase unknown’ [18]. SNPs were imported into RStudio v1.2.1335 [69] and analyzed with the RQTL package using Haldane mapping function [70]. Linkage groups were formed using *formLinkageGroups()* based on a minimum LOD of 5 and a maximum recombination fraction of 0.3. Since markers were present in both phases, two symmetrical sets of linkage groups were generated as expected. After discarding one set, duplicate markers (= identical genotype information) were identified using the RQTL function - *findDupMarkers()* and eliminated when relating to the same SNP, leaving 1023 markers. The marker order in each linkage group was determined using the *orderMarkers()* command. Subsequently, all linkage groups were manually searched for gaps > 20 cM, and to fill in those gaps, the *tryallpositions()* function was applied using 3975 previously excluded markers. After initial linkage map construction, 394 additional markers were manually added that had earlier been filtered out as duplicates. These markers were identical in genotype to markers already in the linkage map but physically mapped to a different genomic scaffold in the Fvar_1.2 genome [55] and thus extended physical coverage. Thus, we had a total of 1417 markers in our final linkage map.

### Comparison to *F. varia* Genome

A nucleotide BLAST (nBLAST) search [71] was performed for the sequence associated with each SNP marker in the *F. varia* genome assembly Fvar_v1.2 (GenBank assembly accession: GCA_011392965.1). An E-value threshold of 1e-50 was used, which returned at least one match for 1404 (of 1417 total) markers. The other markers were considered to be located in sequences that are missing from Fvar_v1.2. The best match of each sequence to a scaffold was considered for assigning scaffolds to linkage groups. When markers from different linkage groups matched the same scaffold, we assigned the scaffold to only one linkage group based on the following rules. First, scaffolds were assigned to a linkage group based on a simple majority rule when the number of matching markers differed between linkage groups. In cases with an equal number of matching markers, the synteny of linkage groups and scaffolds was considered. Still unresolved cases were decided based on the E-value of individual nBLAST matches.

### Comparative visualization of recombination rates

The R package *phytools* [63] was used to create a visual representation of the evolution of recombination rates in the order Hymenoptera by estimating ancestral states using fastAnc() function, based on their phylogeny [60, 72–83] and focusing on species with available genome-wide recombination rate estimates [18–23, 34, 40, 61, 62].

## Supplementary Information

**Additional file 1: Figure S1.** Alignment of all linkage groups markers to *F. varia* genome assembly (F_var1.2).

**Additional file 2: Figure S2.** Relation between physical size and recombination rates of linkage groups.

**Additional file 3. File S1.** R-codes, macros, and terminal command lines used in this study.

**Additional file 4: File S2.** R-Codes, macros, and terminal command lines used in this study. Annotated text file that describe all the codes used at different analyses performed in this genetic map study.

**Additional file 5: File S3.** Divergence time data for phylogenetic tree. Newick tree format file containing approximate divergence time between species. All the data is obtained from references cited in Fig. 3 legend.

**Additional file 6: File S4.** Recombination rate data for phylogenetic tree. Recombination rate data used in phylogenetic tree. All the data is obtained from references cited in Fig. 3 legend.

**Additional file 7: Table S1.** Summary statistics of sequenced reads. The text file shows high-quality read counts for each male bee sample, % alignment to the reference, and proportion of the genotypes.

**Additional file 8: Table S2.** Sequence alignment output of markers to the genome assembly. BLAST output of mapped SNPs (with linkage group placement info) against *F. varia* genome assembly (F_var1.2).

## Data Availability

The raw sequencing data and draft genome assembly reported in this paper have been deposited in the NCBI BioProject accession number PRJNA668370 (https://www.ncbi.nlm.nih.gov/bioproject/?term=PRJNA668370). In-text cited supplementary data (Figure S1, Figure S2, File S1, File S2, Table S1, and Table S2), R codes/macros (File S2), and data to generate phylogeny (File S3 and File S4) can be accessed as online supplementary materials and are also available to download at Zenodo database (10.5281/zenodo.4638539) .

## Abbreviations

cM: Centimorgan
SNP: Single nucleotide polymorphism
VCF: Variant calling format
nBLAST: Nucleotide basic local alignment search tool
LG: Linkage group
