# Supplementary material for: Recombination mapping of the Brazilian stingless bee Frieseomelitta varia confirms high recombination rates in social hymenoptera
Source: BMC Genomics. 2021 Sep 18;22:673. doi: 10.1186/s12864-021-07987-3 (PMC8449902; doi:10.1186/s12864-021-07987-3)
Supplement: Supplementary file 1 — Additional file 1: Figure S1. Alignment of all linkage groups markers to F. varia genome assembly (F_var1.2). [file 12864_2021_7987_MOESM1_ESM.pdf]

Figure S1: Alignment of all linkage groups markers to *F. varia* genome assembly (F_var1.2). BLAST results show synteny between linkage groups to genome scaffolds. Some markers placed at the same position in the linkage map but returned hit to different scaffolds have denoted as an extra line. A marker without a BLAST hit points towards white space. Local ordering of some scaffolds shows hits more than once at some locations, denoted by an asterisk(*). Some scaffolds matched to more than one linkage group, and they have been highlighted with light blue text color and a hashtag superscript ($^{\#}$) for better visualization. Blue boxes are drawn to represent the matching genome scaffolds, but their size is only an approximate indicator of scaffold size.

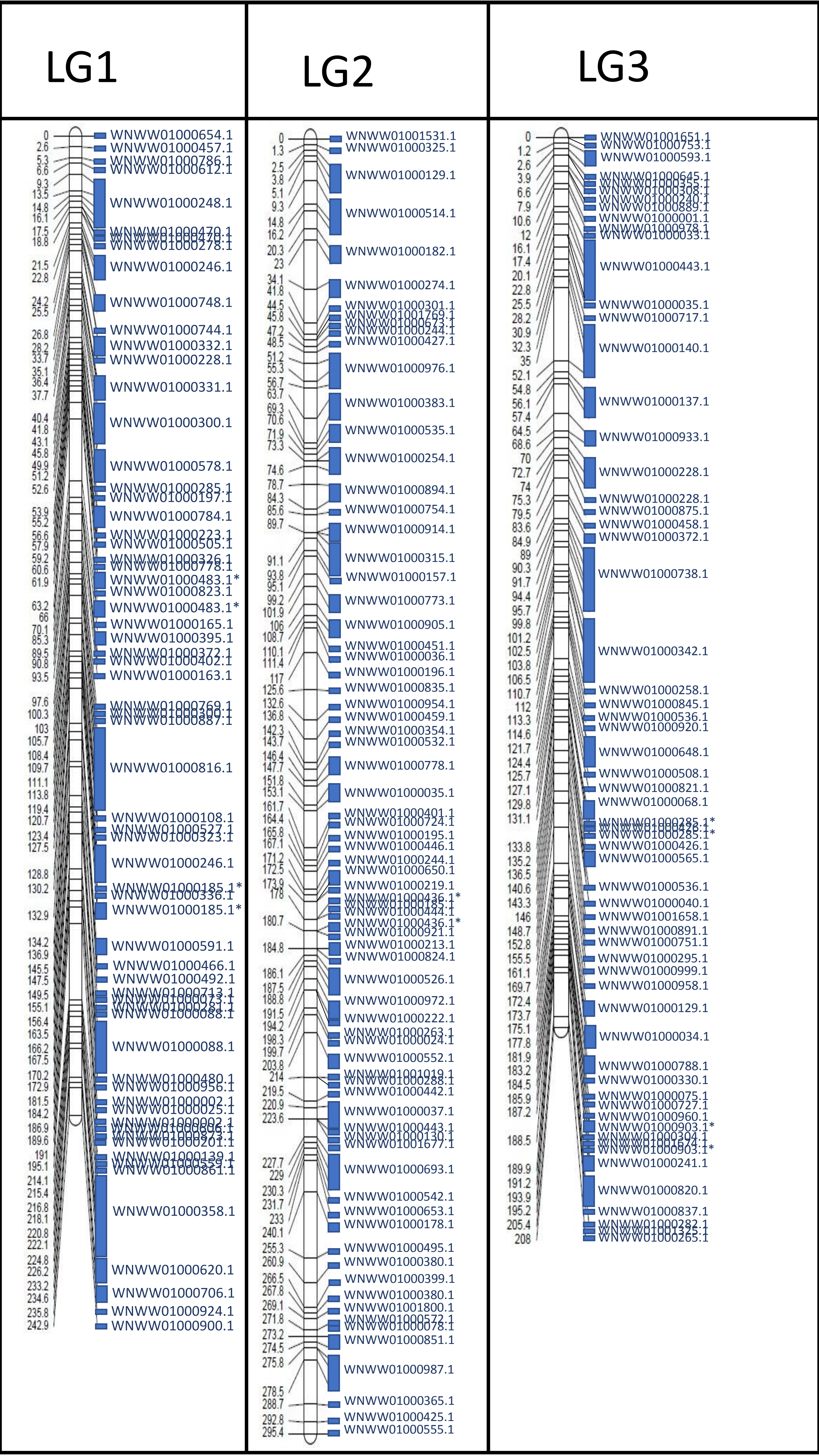

LG1
LG2
LG3
WNWW01000654.1
WNWW01000457.1
WNWW01000612.1
WNWW01000248.1
WNWW01000278.1
WNWW01000246.1
WNWW01000748.1
WNWW01000744.1
WNWW01000332.1
WNWW01000228.1
WNWW01000331.1
WNWW01000300.1
WNWW01000578.1
WNWW01000197.1
WNWW01000784.1
WNWW01000505.1
WNWW01000778.1
WNWW01000483.1*
WNWW01000823.1
WNWW01000483.1*
WNWW01000165.1
WNWW01000395.1
WNWW01000402.1
WNWW01000163.1
WNWW01000887.1
WNWW01000816.1
WNWW01000108.1
WNWW01000323.1
WNWW01000246.1
WNWW01000336.1
WNWW01000185.1*
WNWW01000591.1
WNWW01000466.1
WNWW01000492.1
WNWW01000088.1
WNWW01000088.1
WNWW01000956.1
WNWW01000025.1
WNWW01000201.1
WNWW01000861.1
WNWW01000358.1
WNWW01000620.1
WNWW01000706.1
WNWW01000924.1
WNWW01000900.1
WNWW01001531.1
WNWW01000325.1
WNWW01000129.1
WNWW01000514.1
WNWW01000182.1
WNWW01000274.1
WNWW01000301.1
WNWW01000244.1
WNWW01000427.1
WNWW01000976.1
WNWW01000383.1
WNWW01000535.1
WNWW01000254.1
WNWW01000894.1
WNWW01000754.1
WNWW01000914.1
WNWW01000315.1
WNWW01000157.1
WNWW01000773.1
WNWW01000905.1
WNWW01000451.1
WNWW01000036.1
WNWW01000196.1
WNWW01000835.1
WNWW01000954.1
WNWW01000459.1
WNWW01000354.1
WNWW01000532.1
WNWW01000778.1
WNWW01000035.1
WNWW01000401.1
WNWW01000724.1
WNWW01000195.1
WNWW01000446.1
WNWW01000244.1
WNWW01000650.1
WNWW01000219.1
WNWW01000444.1
WNWW01000921.1
WNWW01000213.1
WNWW01000824.1
WNWW01000526.1
WNWW01000972.1
WNWW01000222.1
WNWW01000024.1
WNWW01000552.1
WNWW01000288.1
WNWW01000442.1
WNWW01000037.1
WNWW01000443.1
WNWW01001677.1
WNWW01000693.1
WNWW01000542.1
WNWW01000653.1
WNWW01000178.1
WNWW01000495.1
WNWW01000380.1
WNWW01000399.1
WNWW01000380.1
WNWW01001800.1
WNWW01000078.1
WNWW01000851.1
WNWW01000987.1
WNWW01000365.1
WNWW01000425.1
WNWW01000555.1
WNWW01001651.1
WNWW01000753.1
WNWW01000593.1
WNWW01000889.1
WNWW01000001.1
WNWW01000033.1
WNWW01000443.1
WNWW01000035.1
WNWW01000717.1
WNWW01000140.1
WNWW01000137.1
WNWW01000933.1
WNWW01000228.1
WNWW01000228.1
WNWW01000875.1
WNWW01000458.1
WNWW01000372.1
WNWW01000738.1
WNWW01000342.1
WNWW01000258.1
WNWW01000845.1
WNWW01000536.1
WNWW01000920.1
WNWW01000648.1
WNWW01000508.1
WNWW01000821.1
WNWW01000068.1
WNWW01000285.1*
WNWW01000426.1
WNWW01000565.1
WNWW01000536.1
WNWW01000040.1
WNWW01001658.1
WNWW01000891.1
WNWW01000751.1
WNWW01000295.1
WNWW01000999.1
WNWW01000958.1
WNWW01000129.1
WNWW01000034.1
WNWW01000788.1
WNWW01000330.1
WNWW01000075.1
WNWW01000727.1
WNWW01000960.1
WNWW01000903.1*
WNWW01000903.1*
WNWW01000241.1
WNWW01000820.1
WNWW01000837.1
WNWW01000265.1

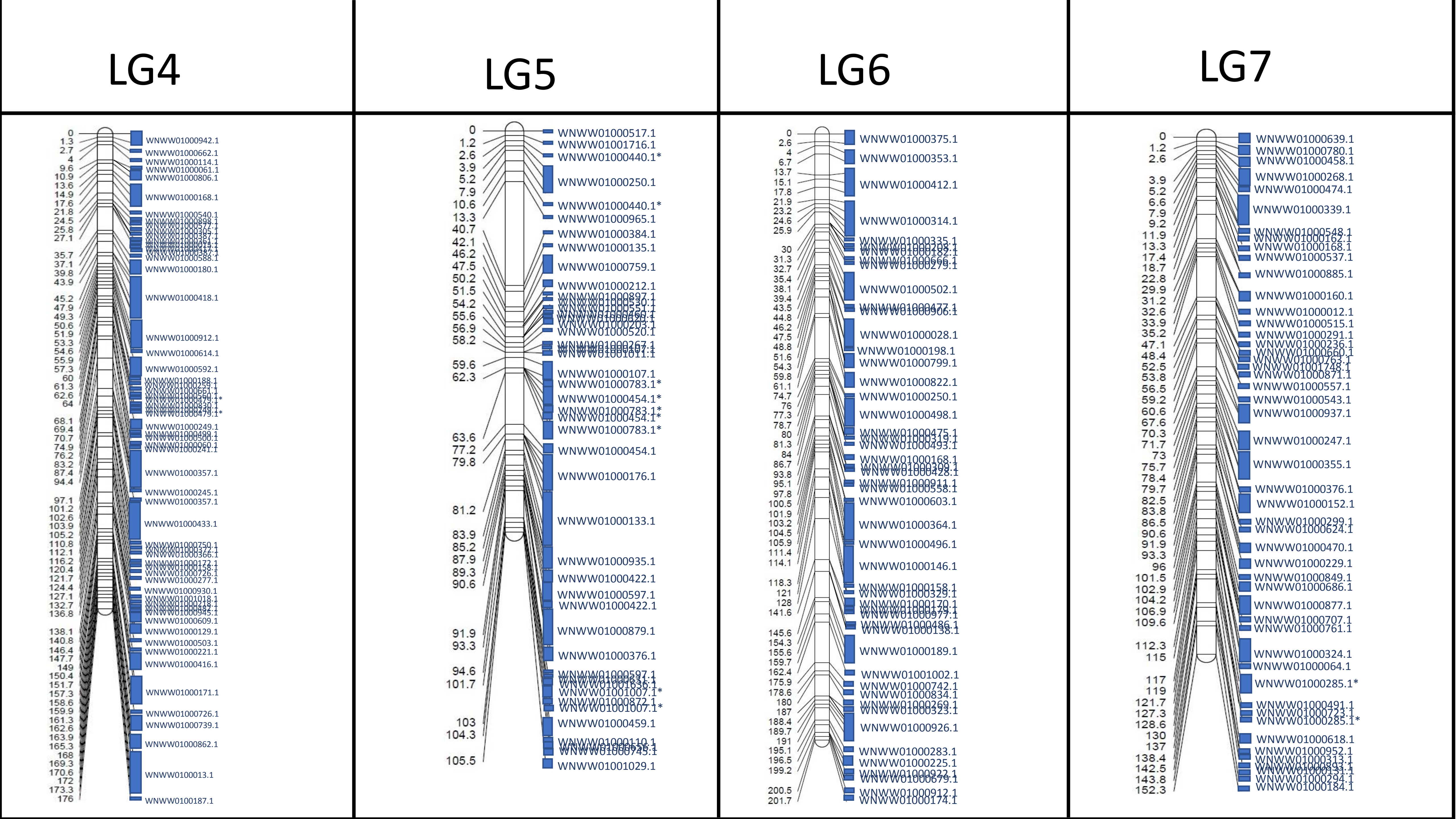
LG4
LG5
LG6
LG7

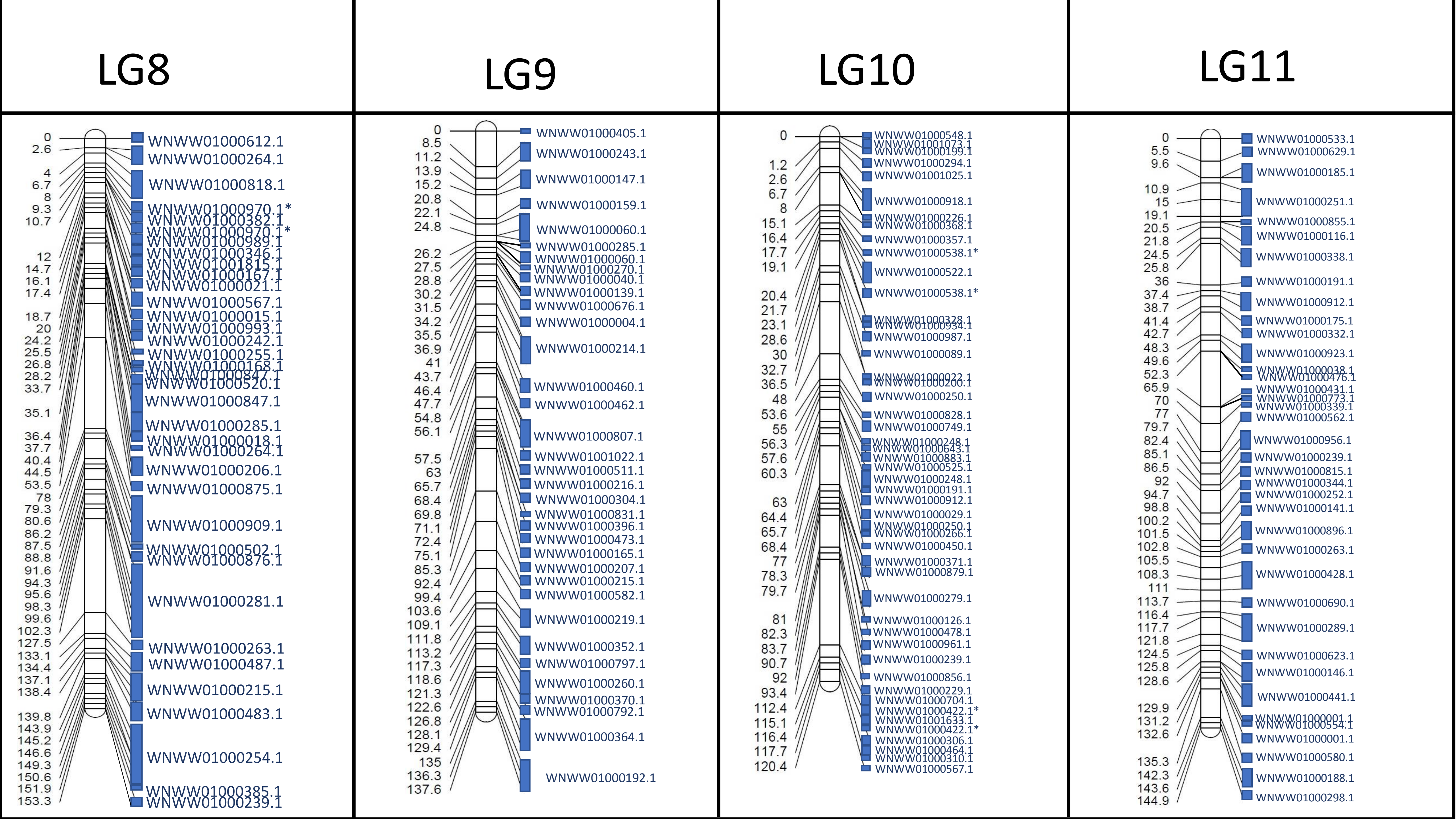
LG8
LG9
LG10
LG11
0
2.6
4
6.7
8
9.3
10.7
12
14.7
16.1
17.4
18.7
20
24.2
25.5
26.8
28.2
33.7
35.1
36.4
37.7
40.4
44.5
53.5
78
79.3
80.6
86.2
87.5
88.8
91.6
94.3
95.6
98.3
99.6
102.3
127.5
133.1
134.4
137.1
138.4
139.8
143.9
145.2
146.6
149.3
150.6
151.9
153.3
WNWW01000612.1
WNWW01000264.1
WNWW01000818.1
WNWW01000970.1*
WNWW01000382.1
WNWW01000970.1*
WNWW01000989.1
WNWW01000346.1
WNWW01001815.1
WNWW01000167.1
WNWW01000021.1
WNWW01000567.1
WNWW01000015.1
WNWW01000993.1
WNWW01000242.1
WNWW01000255.1
WNWW01000168.1
WNWW01000847.1
WNWW01000520.1
WNWW01000847.1
WNWW01000285.1
WNWW01000018.1
WNWW01000264.1
WNWW01000206.1
WNWW01000875.1
WNWW01000909.1
WNWW01000502.1
WNWW01000876.1
WNWW01000281.1
WNWW01000263.1
WNWW01000487.1
WNWW01000215.1
WNWW01000483.1
WNWW01000254.1
WNWW01000385.1
WNWW01000239.1
0
8.5
11.2
13.9
15.2
20.8
22.1
24.8
26.2
27.5
28.8
30.2
31.5
34.2
35.5
36.9
41
43.7
46.4
47.7
54.8
56.1
57.5
63
65.7
68.4
69.8
71.1
72.4
75.1
85.3
92.4
99.4
103.6
109.1
111.8
113.2
117.3
118.6
121.3
122.6
126.8
128.1
129.4
135
136.3
137.6
WNWW01000405.1
WNWW01000243.1
WNWW01000147.1
WNWW01000159.1
WNWW01000060.1
WNWW01000285.1
WNWW01000060.1
WNWW01000270.1
WNWW01000040.1
WNWW01000139.1
WNWW01000676.1
WNWW01000004.1
WNWW01000214.1
WNWW01000460.1
WNWW01000462.1
WNWW01000807.1
WNWW01001022.1
WNWW01000511.1
WNWW01000216.1
WNWW01000304.1
WNWW01000831.1
WNWW01000396.1
WNWW01000473.1
WNWW01000165.1
WNWW01000207.1
WNWW01000215.1
WNWW01000582.1
WNWW01000219.1
WNWW01000352.1
WNWW01000797.1
WNWW01000260.1
WNWW01000370.1
WNWW01000792.1
WNWW01000364.1
WNWW01000192.1
0
1.2
2.6
6.7
8
15.1
16.4
17.7
19.1
20.4
21.7
23.1
28.6
30
32.7
36.5
48
53.6
55
56.3
57.6
60.3
63
64.4
65.7
68.4
77
78.3
79.7
81
82.3
83.7
90.7
92
93.4
112.4
115.1
116.4
117.7
120.4
WNWW01000548.1
WNWW01001073.1
WNWW01000199.1
WNWW01000294.1
WNWW01001025.1
WNWW01000918.1
WNWW01000226.1
WNWW01000368.1
WNWW01000357.1
WNWW01000538.1*
WNWW01000522.1
WNWW01000538.1*
WNWW01000328.1
WNWW01000934.1
WNWW01000987.1
WNWW01000089.1
WNWW01000022.1
WNWW01000200.1
WNWW01000250.1
WNWW01000828.1
WNWW01000749.1
WNWW01000248.1
WNWW01000643.1
WNWW01000883.1
WNWW01000525.1
WNWW01000248.1
WNWW01000191.1
WNWW01000912.1
WNWW01000029.1
WNWW01000250.1
WNWW01000266.1
WNWW01000450.1
WNWW01000371.1
WNWW01000879.1
WNWW01000279.1
WNWW01000126.1
WNWW01000478.1
WNWW01000961.1
WNWW01000239.1
WNWW01000856.1
WNWW01000229.1
WNWW01000704.1
WNWW01000422.1*
WNWW01001633.1
WNWW01000422.1*
WNWW01000306.1
WNWW01000464.1
WNWW01000310.1
WNWW01000567.1
0
5.5
9.6
10.9
15
19.1
20.5
21.8
24.5
25.8
36
37.4
38.7
41.4
42.7
48.3
49.6
52.3
65.9
70
77
79.7
82.4
85.1
86.5
92
94.7
98.8
100.2
101.5
102.8
105.5
108.3
111
113.7
116.4
117.7
121.8
124.5
125.8
128.6
129.9
131.2
132.6
135.3
142.3
143.6
144.9
WNWW01000533.1
WNWW01000629.1
WNWW01000185.1
WNWW01000251.1
WNWW01000855.1
WNWW01000116.1
WNWW01000338.1
WNWW01000191.1
WNWW01000912.1
WNWW01000175.1
WNWW01000332.1
WNWW01000923.1
WNWW01000038.1
WNWW01000476.1
WNWW01000431.1
WNWW01000773.1
WNWW01000339.1
WNWW01000562.1
WNWW01000956.1
WNWW01000239.1
WNWW01000815.1
WNWW01000344.1
WNWW01000252.1
WNWW01000141.1
WNWW01000896.1
WNWW01000263.1
WNWW01000428.1
WNWW01000690.1
WNWW01000289.1
WNWW01000623.1
WNWW01000146.1
WNWW01000441.1
WNWW01000001.1
WNWW01000554.1
WNWW01000001.1
WNWW01000580.1
WNWW01000188.1
WNWW01000298.1

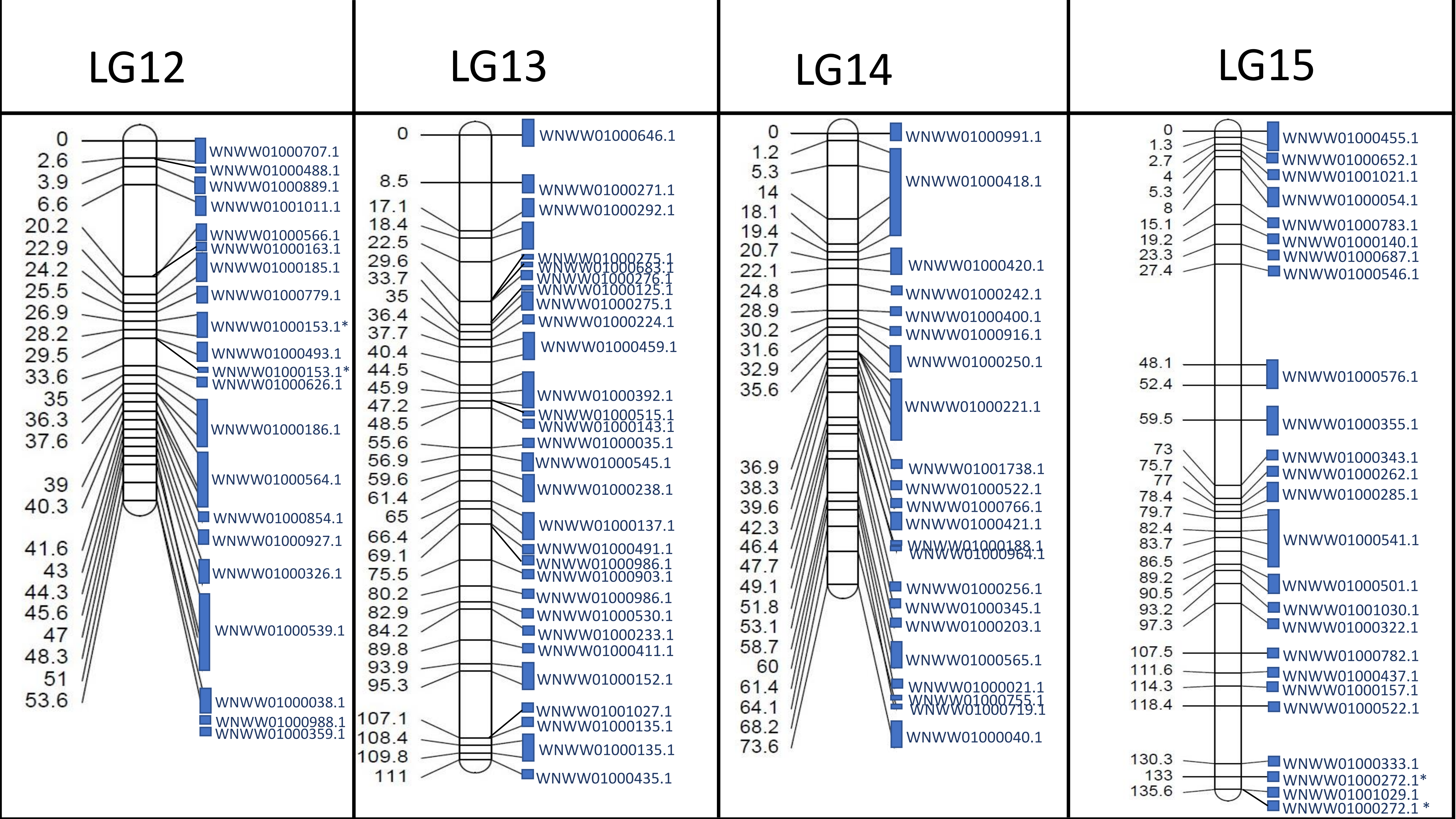
LG12
0
2.6
3.9
6.6
20.2
22.9
24.2
25.5
26.9
28.2
29.5
33.6
35
36.3
37.6
39
40.3
41.6
43
44.3
45.6
47
48.3
51
53.6
WNWW01000707.1
WNWW01000488.1
WNWW01000889.1
WNWW01001011.1
WNWW01000566.1
WNWW01000163.1
WNWW01000185.1
WNWW01000779.1
WNWW01000153.1*
WNWW01000493.1
WNWW01000153.1*
WNWW01000626.1
WNWW01000186.1
WNWW01000564.1
WNWW01000854.1
WNWW01000927.1
WNWW01000326.1
WNWW01000539.1
WNWW01000038.1
WNWW01000988.1
WNWW01000359.1
LG13
0
8.5
17.1
18.4
22.5
29.6
33.7
35
36.4
37.7
40.4
44.5
45.9
47.2
48.5
55.6
56.9
59.6
61.4
65
66.4
69.1
75.5
80.2
82.9
84.2
89.8
93.9
95.3
107.1
108.4
109.8
111
WNWW01000646.1
WNWW01000271.1
WNWW01000292.1
WNWW01000275.1
WNWW01000683.1
WNWW01000276.1
WNWW01000125.1
WNWW01000275.1
WNWW01000224.1
WNWW01000459.1
WNWW01000392.1
WNWW01000515.1
WNWW01000143.1
WNWW01000035.1
WNWW01000545.1
WNWW01000238.1
WNWW01000137.1
WNWW01000491.1
WNWW01000986.1
WNWW01000903.1
WNWW01000986.1
WNWW01000530.1
WNWW01000233.1
WNWW01000411.1
WNWW01000152.1
WNWW01001027.1
WNWW01000135.1
WNWW01000135.1
WNWW01000435.1
LG14
0
1.2
5.3
14
18.1
19.4
20.7
22.1
24.8
28.9
30.2
31.6
32.9
35.6
36.9
38.3
39.6
42.3
46.4
47.7
49.1
51.8
53.1
58.7
60
61.4
64.1
68.2
73.6
WNWW01000991.1
WNWW01000418.1
WNWW01000420.1
WNWW01000242.1
WNWW01000400.1
WNWW01000916.1
WNWW01000250.1
WNWW01000221.1
WNWW01001738.1
WNWW01000522.1
WNWW01000766.1
WNWW01000421.1
WNWW01000188.1
WNWW01000964.1
WNWW01000256.1
WNWW01000345.1
WNWW01000203.1
WNWW01000565.1
WNWW01000021.1
WNWW01000755.1
WNWW01000719.1
WNWW01000040.1
LG15
0
1.3
2.7
4
5.3
8
15.1
19.2
23.3
27.4
48.1
52.4
59.5
73
75.7
77
78.4
79.7
82.4
83.7
86.5
89.2
90.5
93.2
97.3
107.5
111.6
114.3
118.4
130.3
133
135.6
WNWW01000455.1
WNWW01000652.1
WNWW01001021.1
WNWW01000054.1
WNWW01000783.1
WNWW01000140.1
WNWW01000687.1
WNWW01000546.1
WNWW01000576.1
WNWW01000355.1
WNWW01000343.1
WNWW01000262.1
WNWW01000285.1
WNWW01000541.1
WNWW01000501.1
WNWW01001030.1
WNWW01000322.1
WNWW01000782.1
WNWW01000437.1
WNWW01000157.1
WNWW01000522.1
WNWW01000333.1
WNWW01000272.1*
WNWW01001029.1
WNWW01000272.1 *

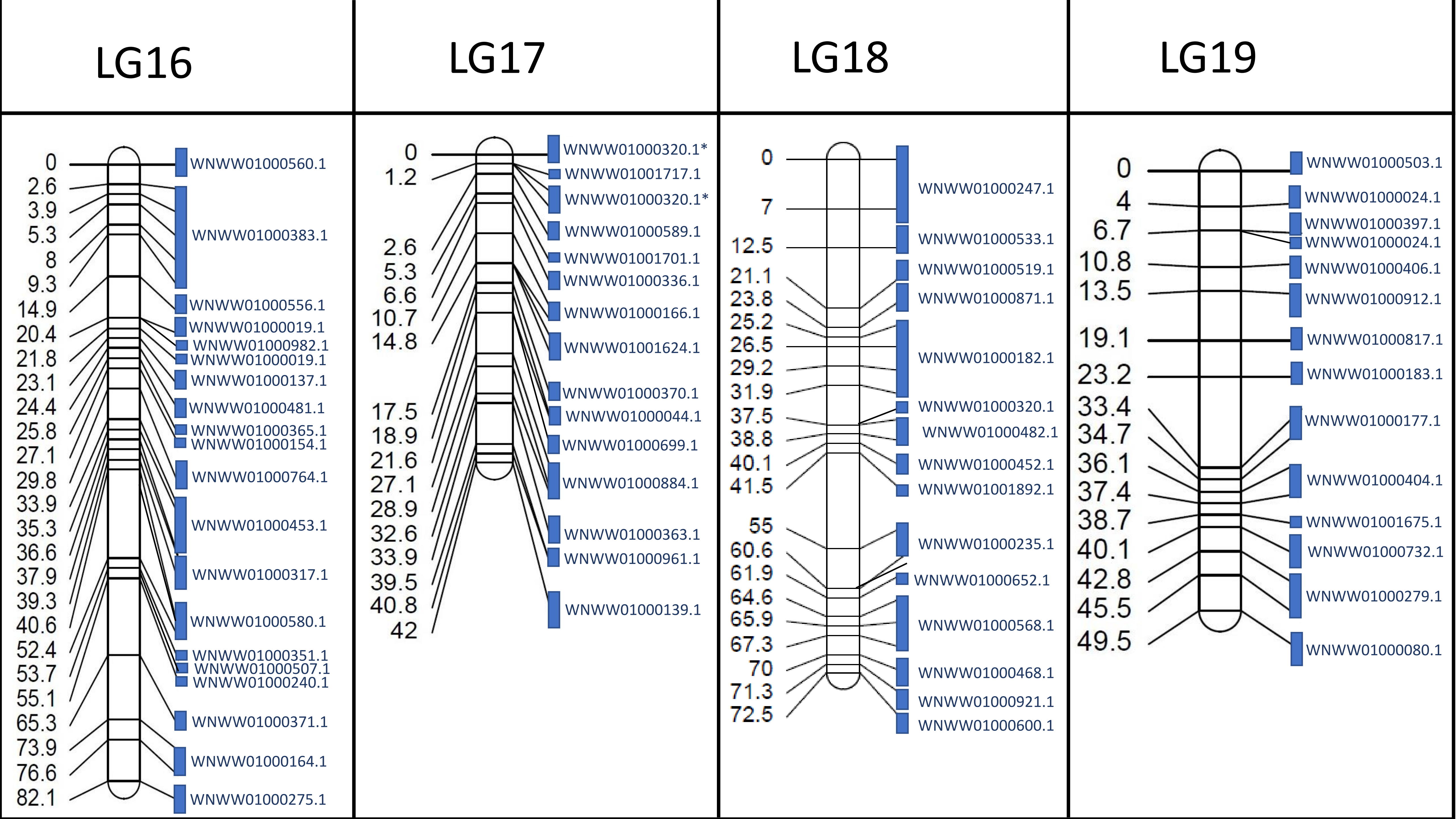
LG16
LG17
LG18
LG19
0
2.6
3.9
5.3
8
9.3
14.9
20.4
21.8
23.1
24.4
25.8
27.1
29.8
33.9
35.3
36.6
37.9
39.3
40.6
52.4
53.7
55.1
65.3
73.9
76.6
82.1
WNWW01000560.1
WNWW01000383.1
WNWW01000556.1
WNWW01000019.1
WNWW01000982.1
WNWW01000019.1
WNWW01000137.1
WNWW01000481.1
WNWW01000365.1
WNWW01000154.1
WNWW01000764.1
WNWW01000453.1
WNWW01000317.1
WNWW01000580.1
WNWW01000351.1
WNWW01000507.1
WNWW01000240.1
WNWW01000371.1
WNWW01000164.1
WNWW01000275.1
0
1.2
2.6
5.3
6.6
10.7
14.8
17.5
18.9
21.6
27.1
28.9
32.6
33.9
39.5
40.8
42
WNWW01000320.1*
WNWW01001717.1
WNWW01000320.1*
WNWW01000589.1
WNWW01001701.1
WNWW01000336.1
WNWW01000166.1
WNWW01001624.1
WNWW01000370.1
WNWW01000044.1
WNWW01000699.1
WNWW01000884.1
WNWW01000363.1
WNWW01000961.1
WNWW01000139.1
0
7
12.5
21.1
23.8
25.2
26.5
29.2
31.9
37.5
38.8
40.1
41.5
55
60.6
61.9
64.6
65.9
67.3
70
71.3
72.5
WNWW01000247.1
WNWW01000533.1
WNWW01000519.1
WNWW01000871.1
WNWW01000182.1
WNWW01000320.1
WNWW01000482.1
WNWW01000452.1
WNWW01001892.1
WNWW01000235.1
WNWW01000652.1
WNWW01000568.1
WNWW01000468.1
WNWW01000921.1
WNWW01000600.1
0
4
6.7
10.8
13.5
19.1
23.2
33.4
34.7
36.1
37.4
38.7
40.1
42.8
45.5
49.5
WNWW01000503.1
WNWW01000024.1
WNWW01000397.1
WNWW01000024.1
WNWW01000406.1
WNWW01000912.1
WNWW01000817.1
WNWW01000183.1
WNWW01000177.1
WNWW01000404.1
WNWW01001675.1
WNWW01000732.1
WNWW01000279.1
WNWW01000080.1
