# Supplementary material for: Recombination mapping of the Brazilian stingless bee Frieseomelitta varia confirms high recombination rates in social hymenoptera
Source: BMC Genomics. 2021 Sep 18;22:673. doi: 10.1186/s12864-021-07987-3 (PMC8449902; doi:10.1186/s12864-021-07987-3)
Supplement: Supplementary file 2 — Additional file 2: Figure S2. Relation between physical size and recombination rates of linkage groups. [file 12864_2021_7987_MOESM2_ESM.pdf]

Figure S2: Relation between physical size and recombination rates of linkage groups.

Spearman's correlation shows a negative trend between physical size to the recombination rate of linkage groups, although not statistically significant ($R = -0.38$, $p = 0.11$). This trend was expected since longer linkage groups will have less crossover per Mb and vice versa if we assume one obligate crossover per chromosome arm. The dark line shows a linear relationship with the shaded area represents a 95% confidence interval.

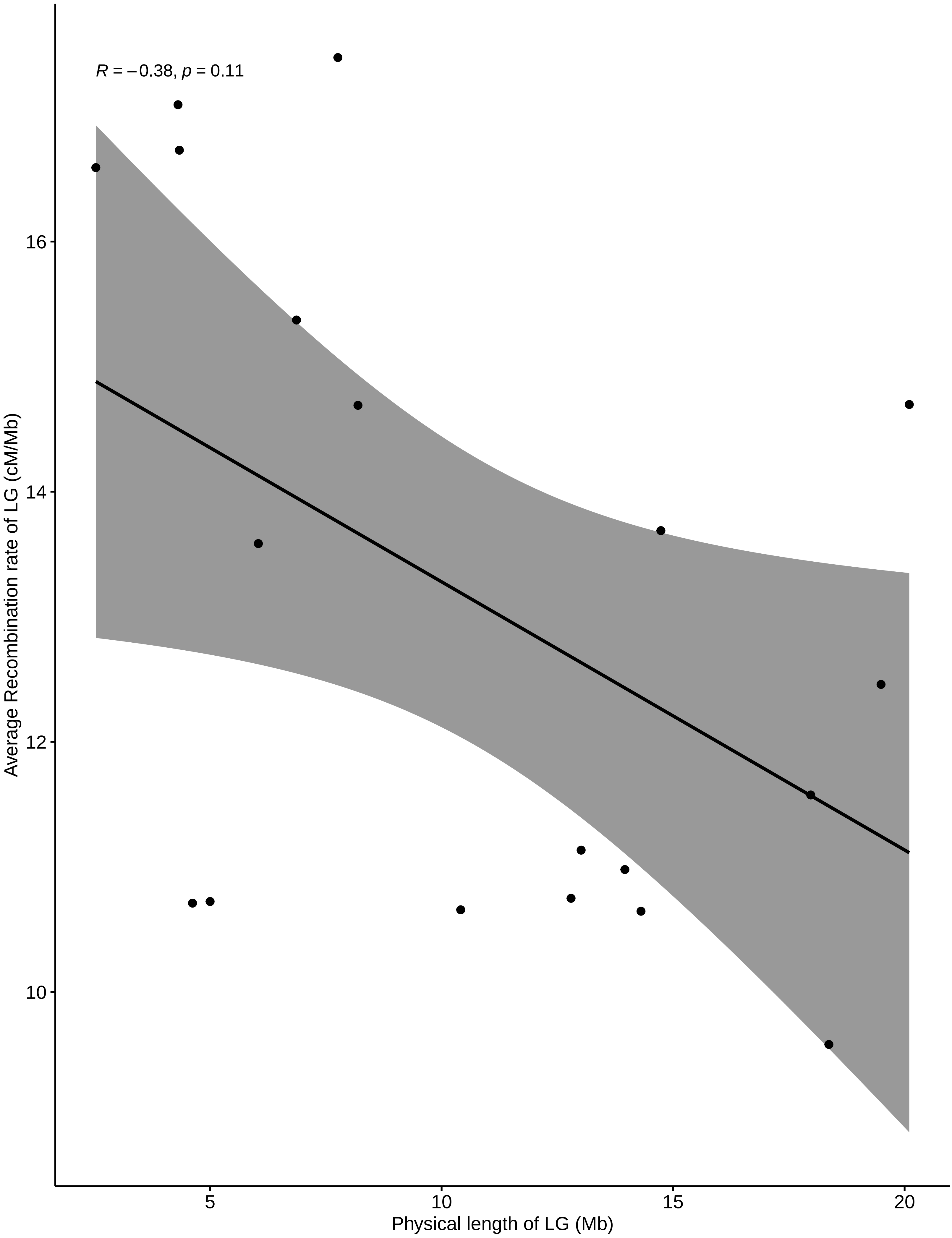

R = −0.38, p = 0.11
Average Recombination rate of LG (cM/Mb)
16
14
12
10
5
10
15
20
Physical length of LG (Mb)
